# Microbial interventions are an easier alternative to engineer higher organisms

**DOI:** 10.1111/1751-7915.13682

**Published:** 2020-10-18

**Authors:** F. Dean Keck, Karen M. Polizzi

**Affiliations:** ^1^ Department of Chemical Engineering Imperial College London London SW7 2AZ UK; ^2^ Imperial College Centre for Synthetic Biology Imperial College London London SW7 2AZ UK

## Abstract

Advances in synthetic biology have made microbes easier to engineer than ever before. However, synthetic biology in animals and plants has lagged behind. Since it is now known that the phenotype of higher organisms depends largely on their microbiota, we propose that this is an easier route to achieving synthetic biology applications in these organisms.

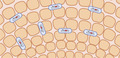

A transition from reading to writing biology has blurred the lines between basic science and engineering creating the field of synthetic biology. With an ever‐expanding genetic toolbox, we now manipulate natural biological systems to optimize our anthropocentric activities. From the synthesis of complex aromatic compounds, to the production of safer vaccines, a problem identified may find its solution lying in the metabolism of a single cell. Initially, synthetic biology was largely focused on the production of such commodities at the industrial scale, not only to maximize profitability, but also to minimize energy and resource consumption. Consequently, this paradigm shift has come to alter the notion of a factory by many orders of magnitude and to create a new bridge between the built and natural world, as we employ nature’s evolutionary machinery to address our modern endeavours.

Growth of the genetic toolbox and maturation of synthetic biology as a field has led to speculation about increasingly ambitious applications of writing biology with implications beyond biosynthesis. To date, most applications have been developed using microbes because they are less complex, more well understood and easier to manipulate. Single‐celled organisms can be optimized for production of complicated organic molecules; however, other exploits of genetic engineering will target more ambitious feats and thus require engineering of more than a large monoculture of microbes. Applications of synthetic biology outside of the bioreactor can address such issues as health and longevity, challenges in industrial agriculture and farming, the degradation of natural habitats and the reclamation of limited natural resources.

Scope and scale of these applications provide obvious obstacles to the development of effective biotechnologies, but a more immediate limitation to realizing these technologies is the relative lack of genetic tools and insights which would allow the tinkering and rewiring of more complex organisms such as animals and plants. However, because of the natural intimate interactions between higher eukaryotes and microbes and the effect of these on phenotype, it is our vision that a faster, more tractable route to the engineering animal and plant phenotypes is via engineering their microbiomes.

## The driver: synthetic biology in higher eukaryotes is slow and difficult

The challenges of synthetic biology in higher eukaryotes stem largely from their complexity. It is no accident that many of the initial proof‐of‐concept studies in synthetic biology used *E. coli* as a chassis, and it was only later that logic gates, toggle switches and oscillators were built in cultured animal and plant cells. For example, despite the development of the genetic toggle switch in *E. coli* in 1999 (Gardner *et al*., 2000), it took another 20 years before an analogous system was engineered for a plant cell (Bernabe‐Orts *et al*., 2020), and to date, they still are not routinely applied in whole organisms. Part of this can be attributed to a lack of well‐characterized parts that can be used in designs and a lack of biological understanding at the systems level for higher eukaryotes. Moreover, even with the application of rapid DNA synthesis techniques and automation, the design‐build‐test‐learn (DBTL) cycle in higher eukaryotes is protracted because of slow growth rates—the doubling times for most microbes are in the range of 20–120 min versus ~ 24‐72 h for plant and animal cells in culture and on the order of weeks or month for whole organisms. Thus, even with perfect ‘design rules’ for engineering an organism, the speed of engineering will always be limited by waiting times between the build and test stages. Finally, the complexity of animals and plants also introduces additional factors not encountered when engineering most microbes including a lack of methods for precision, targeted integration of DNA constructs, differentiation of cells into multiple tissue types, each with varying gene expression patterns, and epigenetic mechanisms that add an additional layer of control over gene expression. These factors combine to make direct application of synthetic biology in animals and plants slow and difficult. In contrast, bacteria have a single, circular genome and the absence of post‐transcriptional modifications. Yeast and fungal systems are slightly more complicated with multiple chromosomes and (usually limited) RNA splicing, but still have a single‐cell lifestyle and limited to no differentiation of cell types. These factors combine to limit complexity and reduce the challenges of applying synthetic biology.

## Animals: from zombie ants to twitching worms

**Fig. 1:**
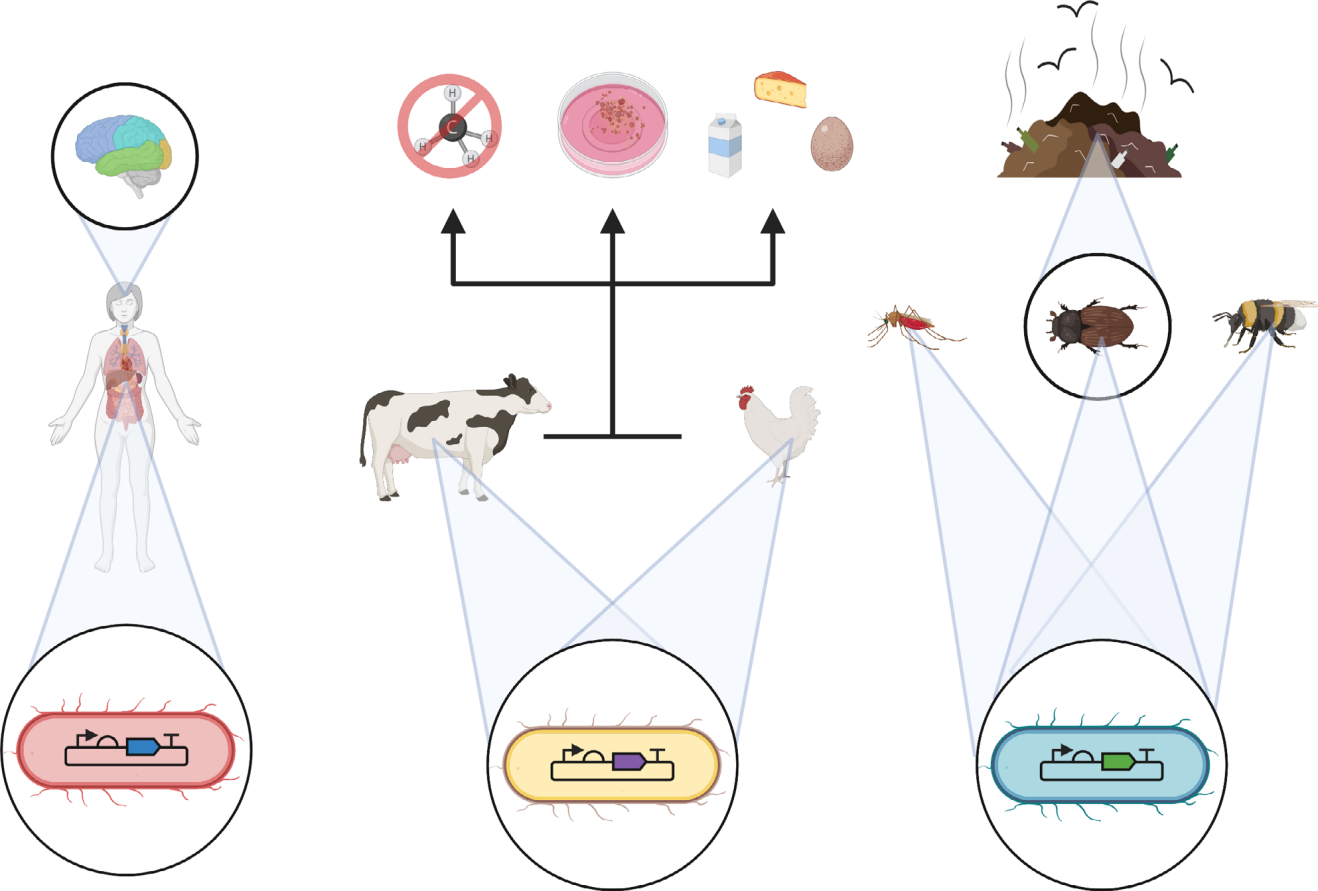
Applications of animal biotechnology that can be achieved through microbiome engineering. Left: Microbiomes can be manipulated to affect human health, behaviour and mood. Middle: Engineering the microbiome of livestock could impact food production (both animal flesh and cultured meat as well as products such as milk and cheese) and affect the environment, e.g. by impacting methane emissions. Right: Insect microbiome engineering affects the roles of insects in disease transmission, entoremediation and food production. Created with BioRender.com.

There are many reasons to engineer animals and not just in science fiction. The potential applications to human and veterinary health are the most often cited—e.g. the ability to engineer replacement or human‐compatible organs tissue for regenerative medicine, vaccine production in animal milk or gene editing as an alternative to gene therapy. There are also myriad applications in food production, bioremediation and ecosystem management that are often overlooked (Figure 1). It is now well known that organisms from the kingdom Animalia live in close collaboration with microbes that colonize them and contribute to their daily existence. The most recent estimates suggest that for a standard ‘reference human’, the number of microbial cells and the number of human cells are about equal (Sender, Fuchs and Milo, 2016). In other words, by cell number, we are half microbes. Various types of microbial niches have been defined including the oral, skin and gut microbiomes, although any accessible tissue will be colonized. Research increasingly suggests that the composition of these microbiomes can affect the health or disease state of an animal through a diverse set of mechanisms (Flórez *et al*., 2015), such as augmenting metabolism, synthesis of toxins or preventing pathogen colonization. Other studies show that animal behaviour can also be influenced directly, e.g. by influencing neurotransmitter activity or reducing stress hormone synthesis (Rohrscheib and Brownlie, 2013). In fact, some researchers have gone as far as to suggest that,

Expression of virtually any host phenotype thus depends to some extent on the presence and taxonomic makeup of host‐associated microbes (Mueller and Sachs, 2015).

Therefore, engineering the microbiome represents a potential way to circumvent some of the challenges of synthetic biology in higher eukaryotes.

The effects of the microbiome on health are the primary area of focus, and studies have shown that the gut microbiome in particular can be involved in the synthesis of vitamins, as well as the metabolism of nutrients (Mohajeri *et al*., 2018). Dysbiosis is associated with inflammatory bowel diseases. Given these are diseases of the gut, it makes mechanistic sense that bacteria can influence their local environment. However, there is emerging evidence that the gut microbiome can impact health more broadly via its influence on the gut/brain axis; for example, there are links between the microbiome and mood/stress responses, as well as overall immune system function (Cryan *et al*., 2019).

There are also intriguing studies on other animal species that point to ability of microbes to influence the behaviour of their hosts to ensure their own survival and reproduction. For example, a particular fungal infection can cause zombie‐like behaviour in ants, where they fall from the canopy, bite into vegetation and become lock‐jawed, allowing the fungus ample opportunity to infect the leaf for the next stage of its life cycle (Hughes *et al*., 2011). Gut microbiota have also been shown to influence mating preference in *Drosophila melanogaster* by changing the levels of circulating sex hormones (Sharona *et al*., 2013). Thus, even biological imperatives like sex and death in animals can be influenced by microbes.

As the importance of microbiomes emerged, it was only natural that scientist would start to try to manipulate them. Initially, efforts at microbiome engineering began with the addition of new naturally occurring microorganisms to an existing microbiome with the aim of inducing colonization. More recent work has explored the potential of using engineered microbes to augment the microbiome with new functions. To date, this has been mostly focused on engineered probiotic strains as therapies (Charbonneau *et al*., 2020) or biosensors (Riglar *et al*., 2017; Rutter *et al*., 2019). However, pushing the concept further, a recent pre‐print showed that host behaviour can be controlled more generally by using the engineered microbes in the gut that themselves respond to externally applied signals to modulate animal gene expression (Gao and Sun, 2020). The paper demonstrated that behaviours such as twitching and metabolism could be controlled in *C. elegans* that were fed *E. coli* engineered to produce inhibitory RNAs against *C. elegans* genes. Chemical inducers were used to control the expression of the inhibitory RNAs within the engineered bacteria, leading to external control of the animal via the microbe. This elegant proof‐of‐concept work could pave the way for broader application of such a strategy within the animal kingdom.

## Plants: terraforming our environment and our food systems

Plants have natural potential for engineering biotechnologies for the agricultural and environmental sectors. Due to their diverse roles within the ecology of terrestrial environments, knowledge of species’ relationships to abiotic and biotic conditions of an ecosystem provides a window into how these traits might be manipulated to address various ecosystem‐scale issues. With a population expected to hit 8 billion people by 2025, synthetic biology poses the potential to rapidly boost productivity within the agricultural sector by engineering plants for increased biomass yields and/or decreased dependence on fertilization. Another application of engineered plants is in the remediation of land contaminated with compounds toxic to both human and environmental health.

**Fig. 2:**
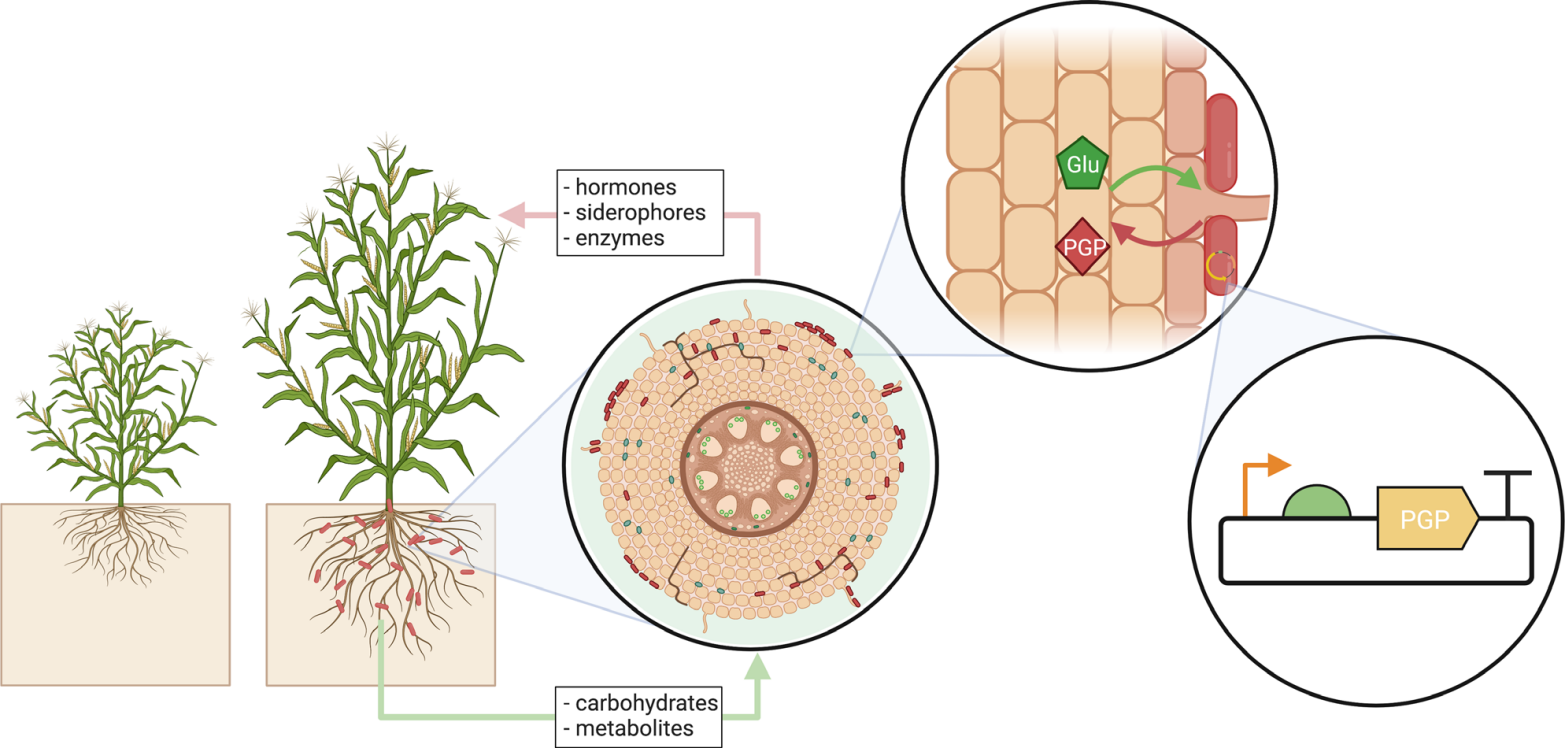
Endophytic bacteria living in close mutualism with roots of a crop plant increase biomass yields when compared to those grown without beneficial endophytes. PGPB can be easily engineered to enhance natural plant growth‐promoting characteristics with simple synthetic biology tools. Here, a bacterium is engineered with an expression plasmid to enhance the biomass potential of its plant host. Created with BioRender.com.

These strategies rely upon the engineering of complex eukaryotic plant cells, slowing down DBTL cycles. However, as with animals, observations of natural relationships between plants and bacteria provide an alternative route to engineering plant phenotypes with relative ease. Many studies have observed the natural, dynamic bacterial community existing within soils, and within plant tissues themselves (endophytes). Bacteria with close association to plant roots are of particular interest for plant health. These plant growth‐promoting bacteria, known as PGPB, have become the subject of interest for the optimization of plant systems for generation of biomass, increase in pathogen tolerance and phytoremediation (Figure 2). One study found that the colonization of Indian Mustard by a strain of bacterium increased biomass yields under both greenhouse and field conditions (Lally *et al*., 2017). Many studies have correlated beneficial growth effects with the bacterial mediation of plant hormones, indole‐3‐acetic acid and ethylene, for instance (Glick, 2014), which are known to be key players in plant growth and development (Shaharoona *et al*., 2007). Other work has engineered inducible fixation of atmospheric nitrogen by cereal endophytes, despite the presence of activity‐limiting soil nitrogen (Ryu *et al*., 2020). As a common limiting nutrient, the ability for more plants to fix their own nitrogen can decrease the environmental burden caused by nitrogen fertilizers, increasing our ability to generate sustainable agriculture. Bacterial production of these compounds has further supported the notion of pushing phenotypical changes through the engineering bacterial metabolism.

Existing examples of using endophytes as alternatives to engineering plant hosts inspire the manipulation of the simpler bacterial genome to elicit effects upon the more complicated plant cell. Much of the current literature observes natural dynamics and proposes tinkering with the expression of bacterial genes, but much of this genetic engineering has yet to be done. We now know through a few studies that similar plant phenotypes can be achieved through the genetic engineering of either plant or bacterial genes involved in the production of similar compounds. By targeting synthesis genes in both *A. thaliana* and its endophytic *P. putida*, one group was able to achieve comparable ethylene production levels between the engineered species (Ravanbakhsh *et al*., 2020). Increasing ethylene production yielded plants that were able to increase shoot concentrations of Fe, Zn and Cu to comparable levels. These data indicate that the indirect engineering of bacterial endophytes can have comparable effects to direct engineering of the plant cell upon the phenotype of a host plant.

Such proof‐of‐concept studies support wider calls in the literature for more attention to microbes living within and among plant hosts for more rapid and impactful bioengineering. For example, rather than attempting to re‐engineer the complex evolutionary machinery of photosynthesis, an alternative is to introduce bacteria producing hormones or enzymes, which will promote biomass accumulation in other ways. Similarly, instead of recombinant expression of metal‐resistant genes in plant cells, it is easier to engineer simpler bacterial systems that can live among these plant cells. Future research should endeavour to elucidate the dynamic relationships between endophytes and plants, and exploit them to engineer novel systems for tackling issues of human and environmental health. We propose that engineering endophytic bacteria is the surest way to quickly and efficiently engineer our food systems and our environment to ensure a sustainable future.

## Summary and future perspective

Foundational research has elucidated the relationship between eukaryotes and their microbiome, showing that the phenotypes of both plants and animals are vastly impacted by microbes. At the same time, synthetic biology is maturing and applications beyond the bioreactor are being proposed. Many of these applications involve the use of animal or plant biotechnologies to address societal challenges in food production, ecosystem health and environmental remediation. Given that undertaking synthetic biology in microbes is orders of magnitude easier than in higher eukaryotes, we propose that future work should focus on maximizing the benefit of this intimate relationship. Within the next 5 years, we predict that the preferred way to engineer animals and plants for biotechnology purposes will be via manipulating their microbiomes, rather than direct manipulation of their own genes.

## References

[mbt213682-bib-0002] Bernabe‐Orts, J.O. , Quijano‐Rubio, A. , Vazquez‐Vilar, M. , Mancheño‐Bonillo, J. , Moles‐Casas, V. , Selma, S. , *et al* (2020) A reversible memory switch for plant synthetic biology based on the phage PhiC31 integration system. Nucleic Acids Res 48: 3379–3394. 32083668 10.1093/nar/gkaa104 PMC7102980

[mbt213682-bib-0003] Charbonneau, M.R. , *et al* (2020) Developing a new class of engineered live bacterial therapeutics to treat human diseases. Nat Commun 11: 1–11. 32269218 10.1038/s41467-020-15508-1 PMC7142098

[mbt213682-bib-0004] Cryan, J.F. , O'Riordan, K.J. , Cowan, C.S.M. , Sandhu, K.V. , Bastiaanssen, T.F.S. , Boehme, M. , *et al* (2019) The microbiota‐gut‐brain axis. Physiol Rev 99: 1877–2013. 31460832 10.1152/physrev.00018.2018

[mbt213682-bib-0005] Flórez, L.V. , Biedermann, P.H.W. , Engl, T. , and Kaltenpoth, M. (2015) Defensive symbioses of animals with prokaryotic and eukaryotic microorganisms. Natural Product Rep 32: 904–936. 10.1039/c5np00010f 25891201

[mbt213682-bib-0006] Gao, B. , and Sun, Q. (2020) Programming animal physiology and behaviors through engineered bacteria. bioRxiv 2020.08.15.232637.

[mbt213682-bib-0007] Gardner, T.S. , Cantor, C.R. , and Collins, J.J. (2000) Construction of a genetic toggle switch in *Escherichia coli* . Nature 403: 339–342. 10659857 10.1038/35002131

[mbt213682-bib-0008] Glick, B.R. (2014) Bacteria with ACC deaminase can promote plant growth and help to feed the world. Microbiol Res 169: 30–39. 24095256 10.1016/j.micres.2013.09.009

[mbt213682-bib-0009] Hughes, D.P. , Andersen, S.B. , Hywel‐Jones, N.L. , Himaman, W. , Billen, J. , and Boomsma, J.J. (2011) Behavioral mechanisms and morphological symptoms of zombie ants dying from fungal infection. BMC Ecol 11: 13. 21554670 10.1186/1472-6785-11-13 PMC3118224

[mbt213682-bib-0010] Lally, R.D. , Galbally, P. , Moreira, A.S. , Spink, J. , Ryan, D. , Germaine, K.J. , and Dowling, D.N. (2017) Application of endophytic *Pseudomonas fluorescens* and a bacterial consortium to *Brassica napus* can increase plant height and biomass under greenhouse and field conditions. Frontiers Plant Sci 8: 2193. 10.3389/fpls.2017.02193 PMC5744461 29312422

[mbt213682-bib-0011] Mohajeri, M.H. , Brummer, R.J.M. , Rastall, R.A. , Weersma, R.K. , Harmsen, H.J.M. , Faas, M. , *et al* (2018) The role of the microbiome for human health: from basic science to clinical applications. Eur J Nutr 57: 1–14. 10.1007/s00394-018-1703-4 PMC5962619 29748817

[mbt213682-bib-0012] Mueller, U.G. , and Sachs, J.L. (2015) Engineering microbiomes to improve plant and animal health. Trends Microbiol 23: 606–617. 26422463 10.1016/j.tim.2015.07.009

[mbt213682-bib-0013] Ravanbakhsh, M. , Kowalchuk, G.A. , and Jousset, A. (2020) Targeted plant hologenome editing for plant trait enhancement. New Phytol (in press). 10.1111/nph.16867 PMC7820966 32772380

[mbt213682-bib-0014] Riglar, D.T. , Giessen, T.W. , Baym, M. , Kerns, S.J. , Niederhuber, M.J. , Bronson, R.T. , *et al* (2017) Engineered bacteria can function in the mammalian gut long‐term as live diagnostics of inflammation. Nat Biotechnol 35: 653–658. 28553941 10.1038/nbt.3879 PMC5658125

[mbt213682-bib-0015] Rohrscheib, C.E. , and Brownlie, J.C. (2013) Microorganisms that manipulate complex animal behaviours by affecting the host’s nervous system. Springer Sci Rev 1: 133–140.

[mbt213682-bib-0016] Rutter, J.W. , *et al* (2019) Detecting changes in the *Caenorhabditis elegans* intestinal environment using an engineered bacterial biosensor. ACS Synthetic Biol 8(12): 2620–2628. 10.1021/acssynbio.9b00166 PMC6929061 31657907

[mbt213682-bib-0017] Ryu, M.H. , Zhang, J. , Toth, T. , Khokhani, D. , Geddes, B.A. , Mus, F. , *et al* (2020) Control of nitrogen fixation in bacteria that associate with cereals. Nat Microbiol 5: 314–330. 31844298 10.1038/s41564-019-0631-2 PMC8634771

[mbt213682-bib-0018] Sender, R. , Fuchs, S. , and Milo, R. (2016) Revised estimates for the number of human and bacteria cells in the body. PLoS Biol 14: 1–14. 10.1371/journal.pbio.1002533 PMC4991899 27541692

[mbt213682-bib-0019] Shaharoona, B. , *et al* (2007) Effectiveness of various *Pseudomonas* spp. and *Burkholderia caryophylli* containing ACC‐deaminase for improving growth and yield of wheat (*Triticum aestivum* L.). J Microbiol Biotechnol 17: 1300–1307. 18051598

[mbt213682-bib-0020] Sharona, G. , *et al* (2013) Commensal bacteria play a role in mating preference of *Drosophila melanogaster* . PNAS 110: 4852. 10.1073/pnas.1009906107 PMC2993361 21041648

